# Allelopathic effects of volatile organic compounds released from *Pinus halepensis* needles and roots

**DOI:** 10.1002/ece3.5390

**Published:** 2019-07-02

**Authors:** Mathieu Santonja, Anne Bousquet‐Mélou, Stéphane Greff, Elena Ormeño, Catherine Fernandez

**Affiliations:** ^1^ Aix Marseille Univ, Avignon Université, CNRS, IRD, IMBE Marseille France

**Keywords:** Aleppo pine, allelopathy, Mediterranean forest, plant–plant interaction, secondary succession, soil microorganisms, terpenes

## Abstract

The Mediterranean region is recognized as a global biodiversity hotspot. However, over the last decades, the cessation of traditional farming in the north part of the Mediterranean basin has given way to strong afforestation leading to occurrence of abandoned agricultural lands colonized by pioneer expansionist species like *Pinus halepensis*. This pine species is known to synthesize a wide range of secondary metabolites, and previous studies have demonstrated strong allelopathic potentialities of its needle and root leachates. *Pinus halepensis* is also recognized to release significant amounts of volatile organic compounds (VOC) with potential allelopathic effects that have never been investigated. In this context, the objectives of the present study were to improve our knowledge about the VOC released from *P. halepensis* needles and roots, determine if these VOC affect the seed germination and root growth of two herbaceous target species (*Lactuca sativa* and *Linum strictum*), and evaluate if soil microorganisms modulate the potential allelopathic effects of these VOC. Thirty terpenes were detected from both, needle and root emissions with β‐caryophyllene as the major volatile. Numerous terpenes, such as β‐caryophyllene, δ‐terpinene, or α‐pinene, showed higher headspace concentrations according to the gradient green needles < senescent needles < needle litter. Seed germination and root growth of the two target species were mainly reduced in presence of *P. halepensis* VOC. In strong contrast with the trend reported with needle leachates in literature, we observed an increasing inhibitory effect of *P. halepensis* VOC with the progress of needle physiological stages (i.e., green needle < senescent needle < needle litter). Surprisingly, several inhibitory effects observed on filter paper were also found or even amplified when natural soil was used as a substrate, highlighting that soil microorganisms do not necessarily limit the negative effects of VOC released by *P. halepensis* on herbaceous target species.

## 1 INTRODUCTION

Plant community organization and dynamics are under the control of biotic processes, particularly plant–plant interactions such as resource competition, facilitation, and allelopathy (Callaway & Walker, 1997). A strong attention has been paid during the last decades to allelopathy, demonstrating the key implication of plant–plant chemical interaction as a driver of plant community structure and ecosystem functioning (Inderjit, Wardle, Karban, & Callaway, 2011; Meiners, Kong, Ladwig, Pisula, & Lang, 2012; Wardle, Nilsson, Gallet, & Zackrisson, 1998). Seed germination and seedling performance are the main life stages usually affected by allelochemicals, and frequent negative allelopathic effects are inhibition of seed germination (Fernandez et al., 2013; Herranz, Ferrandis, Copete, Duro, & Zalacain, 2006), delay of seed germination (Fernandez et al., 2013; Hashoum et al., 2017), and inhibition of seedling growth (Gavinet et al., 2019; Santonja, Le Rouzic, & Thiebaut, 2018) by altering physiological processes (e.g., photosynthesis, nutrient uptake, cell division, or elongation; Inderjit & Duke, 2003). However, the persistence, availability, and biological impacts of the allelochemicals could be modulated by soil microbial communities (Cipollini, Rigsby, & Barto, 2012; Inderjit, 2005). Indeed, by using different substrates such as filter paper, natural or sterilized soils, several studies highlighted the key role played by soil microorganisms which suppressed the potential negative allelopathic effects (Fernandez et al., 2013; Inderjit, 2005; Kaur, Kaur, Kaur, Baldwin, & Inderjit, 2009). In addition to be highly variable among species, the diversity and quantity of allelochemicals produced by a given species and their influence on a target species are strongly dependent on its phenological stage (Fernandez et al., 2009; Hashoum et al., 2017; Santonja, Le Rouzic, et al., 2018). Surprisingly, most of the published allelopathy studies were performed by using only green leaves (or needles) and thus neglected the allelopathic potentialities of chemicals contained in senescent leaves or leaf litter. Hashoum et al. (2017) reported that the germination velocity of two target herbaceous species (*Festuca ovina* L. and *Linum perenne* L.) was inhibited by aqueous extracts of senescent leaves of woody species (*Acer monspessulanum* L., *Cotinus coggygria* Scop., and *Quercus pubescens* Willd.) while their seedling growth was affected by aqueous extracts of green leaves.

Mediterranean plants synthesize a wide variety of specialized metabolites, which help them to cope with summer drought and high radiative stress (Chaves & Escudero, 1999), and are involved in allelopathic interactions (Scognamiglio et al., 2013; Vilà & Sardans, 1999). Strong evidence is thereby accumulating that allelopathy is a key mechanism shaping plant community diversity and dynamics in Mediterranean ecosystems (Alias, Sosa, Escudero, & Chaves, 2006; Ehlers, Charpentier, & Grøndahl, 2013; Fernandez et al., 2013; Hashoum et al., 2017; Herranz et al., 2006). Among Mediterranean trees, *Pinus halepensis* Mill. has been the subject of numerous recent studies because this pine has expanded massively over the last century facilitated by both, forest fires and farmland abandonment (Richardson et al., 2007). As a result, this pioneer and expansionist species has come to dominate the areas of agricultural decline (Gondard, Romane, Aronson, & Shater, 2003), contributing to the homogenization of plant communities in the North Mediterranean area, where it forms dense monospecific mature forests. *Pinus halepensis* produces large quantities of specialized metabolites including phenolics and terpenes (Fernandez et al., 2009, 2016; Macchioni et al., 2003; Pasqualini et al., 2003) which can alter the composition of plant communities (Fernandez et al., 2006, 2013), but also soil microbial communities (Chomel et al., 2014; Santonja, Foucault, et al., 2018) and ecosystem processes (Chomel et al., 2014; Santonja, Baldy, Fernandez, Balesdent, & Gauquelin, 2015; Santonja, Fernandez, Gauquelin, & Baldy, 2015). As most compounds involved in allelopathic interactions are water‐soluble (Reigosa, Sanchez‐Moreiras, & Gonzalez, 1999; Rice, 1984), previous studies have mostly focused on the allelopathic potentialities of *P. halepensis* needle and root leachates. For example, Fernandez et al. (2013) demonstrated a high sensitivity of herbaceous plant species naturally present in fallow farmlands to allelochemicals released from *P. halepensis* green needles, while Nektarios, Economou, and Avgoulas (2005) reported a decreasing inhibitory effect on both, germination and seedling growth of four target herbaceous species according to the gradient green needles > senescent needles > needle litter. However, no previous studies have focused on allelopathic effects driven by volatile organic compounds (VOC) released by *P. halepensis*, despite this pine species releases important amounts of VOC such as terpenes (Ormeño, Fernandez, Bousquet‐Mélou, et al., 2007) which have been reported to exhibit strong inhibitory effects on seed germination and growth of numerous target herbaceous species (AlSaadawi, Arif, & AlRubeaa, 1985; De Martino, Mancini, Almeida, & Feo, 2010). In addition, terpene emissions from plant species are predicted to increase substantially due to a warmer climate and dense vegetation communities (Peñuelas & Staudt, 2010) indicating the need for further research on the role played by these VOC in ecosystem functioning. In this context, it is of prime interest to improve our knowledge about the allelopathic potentialities of *P. halepensis* VOC.

To fill this gap, we performed a laboratory experiment in order to (a) identify the VOC released from *P. halepensis* needles and roots (green needles, senescent needles, needle litter, and roots), (b) determine if these VOC affect seed germination and root growth of two herbaceous target species (*Lactuca sativa* L. and *Linum strictum* L.), (c) test if these VOC and their effects vary according to the organs (needles vs. roots) and the needle physiological stages, and finally (d) evaluate if soil microorganisms modulate the potential allelopathic effects of these VOC by using filter paper and natural soil as substrate.

## 2 MATERIAL AND METHODS

### 2.1 Material collection

The sampling site was located in the Luberon Natural Regional Park, SE France. This site is a secondary succession following abandonment agricultural lands, including different stages of *P. halepensis* colonization from fallow land with few young pines (<5 years old) to old pine forests (>60 years old).

The soil used as substrate for the bioassays was collected outside the zone of influence of *P. halepensis* (i.e., in a fallow without pine), sieved to a mesh size of 2 mm, and kept at room temperature until the start of the experiment. Green needles, senescent needles, needle litter, and roots of *P. halepensis* were collected in a young *P. halepensis* forest (about 10 years old) at the beginning of summer. While green needles, senescent needles, and roots were directly collected on the trees, needle litter of the current year was collected on the ground under the canopy of the corresponding trees. Material from 10 individuals was collected and pooled every 2 days since fresh pine material was renewed every 2 days in order to perform the laboratory experiments, for a total of 110 individuals sampled during the study.

Two herbaceous species were selected as target of *P. halepensis* VOC. Firstly, *L. strictum* as this herbaceous species is naturally present in the first secondary succession stages following abandonment of agricultural lands in the studied area and had been reported to be highly sensitive to green needle leachates (Fernandez et al., 2006, 2013). Secondly, *L. sativa* since this species is known for its sensitivity to allelopathic substances and is frequently used for bioassays (e.g., Bousquet‐Mélou et al., 2005; Fernandez et al., 2006). Seeds of *L. strictum* were collected from wild populations on the study site outside the zone of influence of *P. halepensis* and then stored in a cold chamber at 4°C until the start of the experiment. Seeds of *L. sativa* were purchased in a garden shop (Truffaut, www.truffaut.com).

### 2.2 Laboratory experiments

#### 2.2.1 Allelopathic bioassay with *Pinus halepensis* VOC

**Figure 1:**
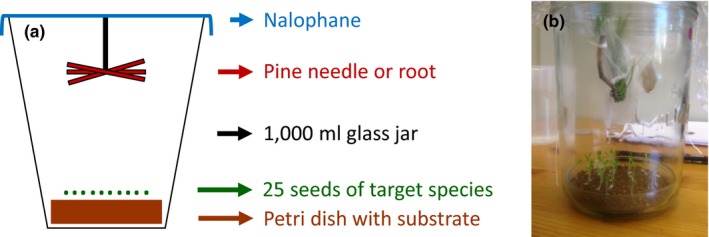
Schematic drawing (a) and picture (b) of the experimental design used to test the effects of the volatile organic compounds released from *Pinus halepensis* needles and roots on seed germination rate and root growth of *Lactuca sativa* and *Linum strictum*. Filter paper or natural soil were used as substrate in the glass Petri dish, and the 1,000 ml glass jar was closed with nalophane to prevent volatile from escaping

This bioassay was conducted with two doses of VOC by suspending 2.5 or 10.0 g (equivalent dry mass, DM) of plant material in a 1 L microcosm (Figure 1). Fresh material was renewed every 2 days. Petri dishes were filled with 50.0 g DM of soil or with two layers of filter paper deposited at the bottom of the microcosm closed with nalophane to prevent VOC from escaping (Figure 1). We compared results from natural soil and filter paper as substrate in order to assess the role of natural soil microbial communities in shaping allelopathic effects (Fernandez et al., 2013; Inderjit, 2005; Kaur et al., 2009). Each Petri dish was sown with 25 seeds of one of the two target species that were watered every 2 days with 2 ml of deionized water for filter paper substrate or 5 ml for soil substrate (Figure 1). Four replicates were performed for each treatment (target species × *Pinus* VOC source × dose × substrate) for a total of 96 microcosms. Bioassays were conducted under natural photoperiod (15 hr: 9 hr day: night regime) and controlled temperature (21 ± 1°C).

Seed germination percentage was calculated as [(number of germinated seeds)/(number of sown seeds)] × 100 (Bousquet‐Mélou et al., 2005; Gavinet et al., 2019; Santonja, Le Rouzic, et al., 2018). Regarding seedling growth, root length (mm) was measured for each individual 5 days after germination (Fernandez et al., 2006, 2013; Hashoum et al., 2017). We calculated a relative allelopathic effect (RAE) index to determine the intensity of the allelopathic effect on seed germination and seedling growth (Gavinet et al., 2019; Hashoum et al., 2017). The RAE index was calculated as (O − C)/C × 100, where O is the value of the plant trait (germination or growth) when a target species is exposed to allelopathic compounds and C the mean value of that trait under control conditions. A negative RAE value indicates an inhibitory effect, whereas a positive RAE value indicates a stimulatory effect.

#### 2.2.2 Allelopathic bioassay with β‐caryophyllene

**Figure 2:**
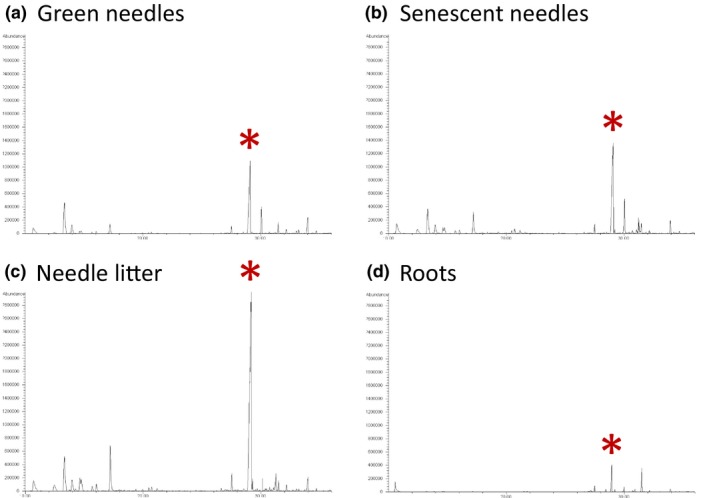
Chromatograms obtained from Solid Phase Micro Extraction (SPME) of the volatile organic compounds released from *Pinus halepensis* green needles (a), senescent needles (b), needle litter (c), and roots (d). Red star indicates β‐caryophyllene (i.e., the main compound released from needles and roots)

In addition to the use of *P. halepensis* material, we tested the effects of β‐caryophyllene, the main VOC released from both, needles and roots (Appendix 1; Figure 2), on the germination and growth of *L. sativa*. β‐caryophyllene (99% high purity standard) was obtained from Sigma‐Aldrich. The experiment was performed with filter paper as substrate (Figure 1). Each Petri dish was sown with 25 seeds that were watered every 2 days with 2 ml of deionized water. The pure compound was dissolved in ethanol (Reigosa & Pazos‐Malvido, 2007) and five different dilutions (10, 50, 100, 500, and 1,000 µM) were prepared. Every 2 days, a piece of filter paper (2 × 1 cm) was impregnated with 1 ml of each solution (or only ethanol for the control treatment), kept few seconds outside the microcosm to evaporate ethanol and then suspended in the 1 L microcosm. Four replicates were performed for each concentration for a total of 24 microcosms. Bioassays were conducted under the same conditions than before (natural photoperiod and controlled temperature: 21 ± 1°C). Germination rate, root growth, and corresponding RAE values were obtained as previously described.

### 2.3 Chemical analysis

Headspace Solid Phase Micro Extraction (SPME) was performed to collect and characterize the chemical composition of volatiles released from *P. halepensis* needles and roots (Jassbi, Zamanizadehnajari, & Baldwin, 2010). A SPME fiber coated with Polydimethylsiloxane/Divinylbenzene (PDMS/DVB, fiber diameter 65 μm, needle size 24 ga, StableFlexTM) was exposed for 4 hr to 10.0 g DM of suspended plant material in each microcosm 1 hr after the pine material was put into the microcosm (Figure 1). The SPME fibers were analyzed on a Hewlett‐Packard GC6890 coupled with a HP5973N Mass Selective Detector and equipped with a HP‐5MS capillary column (30 m × 0.25 mm × 0.25 µm, J&W Scientific). Data were acquired in scan mode from 40 to 300 uma. Retention indexes of compounds were determined relative to Wisconsin Diesel Range Hydrocarbon injection (C8‐C20, Interchim) and compared with those reported in the literature (Adams, 2007). The identification of some terpenes was done by comparison of mass spectra (MS) to those of reference standards (Sigma‐Aldrich®, Appendix 1). Database searches in the NIST 2014 mass spectral library were also conducted to tentatively annotate unidentified components.

### 2.4 Statistical analyses

Statistical analyses were performed with the R software (version 3.3.1). Significance was evaluated in all cases at *p* < 0.05. Normality and homoscedasticity of the residuals of the models were visually checked.

Firstly, differences of seed germination rate and root growth according to target species (*L. sativa *vs.* L. strictum*), substrate type (filter paper vs. natural soil), and their interactions in absence of VOC (i.e., in the control treatments) were assessed using two‐way ANOVAs, followed by Tukey HSD tests for post hoc pairwise comparisons.

Secondly, three‐way ANOVAs, followed by Tukey HSD tests for post hoc pairwise comparisons, were used to test the effects of *P. halepensis* VOC source (green needle, senescent needle, needle litter, and root), dose (low and high), substrate type (filter paper and natural soil), and their interactions on the RAE on seed germination and root growth of the two target species.

Thirdly, Kruskal Wallis tests, followed by post hoc multiple range tests (Fisher's Least Significance Difference), were used to test the effects of β‐caryophyllene concentration on the seed germination and root growth of *L. sativa*.

## 3 RESULTS

### 3.1 Detected volatiles

Solid Phase Micro Extraction fibers were used to trap the emitted VOC from *Pinus* needles and roots (Appendix 1; Figure 2). The GC‐MS analysis revealed that 88% (senescent needles) to 93% (roots) of the detected volatiles were terpenes. Thirty terpenes were detected from both, needle and root emissions, while 20 terpenes were detected only from needles (Appendix 1). In addition, 12 terpenes were detected in emissions from senescent needles and needle litter but not from green needles. Higher headspace concentrations of terpenes were observed in microcosms containing needles compared to roots. Sesquiterpenes from needles were emitted twice more than monoterpenes (58% vs. 32%), whereas the ratio was 72% versus 22% for roots. β‐caryophyllene was the major emitted volatile from both, needles and roots. The other major emitted volatiles from needles were myrcene, δ‐terpinene, and α‐pinene, while α‐pinene, α‐muurolene, and copaene were the other major emitted volatiles from roots. Finally, numerous terpenes, such as β‐caryophyllene, δ‐terpinene, and α‐pinene, showed an increasing concentration according to the gradient green needles < senescent needles < needle litter.

### 3.2 Allelopathic bioassays with *P. halepensis* VOC

Germination rate of *L. sativa* seeds was three times higher than *L. strictum* in the control treatments (*F* = 174.2, *p* < 0.001, Table 1), and germination rate of both species was not affected by substrate type (*p* > 0.05, Table 1). Root growth of *L. sativa* was higher than *L. strictum* (*F* = 131.1, *p* < 0.001, Table 1), and root growth of both species was higher when natural soil was used as substrate compared to filter paper (*F* = 112.5, *p* < 0.001, Table 1).

**Table 1:** Seed germination rate and root growth of *Lactuca sativa* and *Linum strictum* according to substrate type (filter paper vs. natural soil) in the control treatments

	Filter paper	Natural soil
*Lactuca sativa*		
Germination (%)	86.0 ± 3.8	94.0 ± 2.0
Root (mm)	23.2 ± 0.6	32.4 ± 0.7
*Linum strictum*		
Germination (%)	38.0 ± 6.2	30.0 ± 3.8
Root (mm)	18.3 ± 0.6	20.3 ± 0.5

Note

Values are mean ± *SE*.

**Table 2:** Results of three‐way ANOVAs testing for the effects of *Pinus halepensis* VOC source (green needle, senescent needle, needle litter, and root), dose (low vs. high), substrate type (filter paper vs. natural soil), and their interactions on the relative allelopathic effect (RAE) on seed germination rate and root growth of the two target species (*Lactuca sativa* and *Linum strictum*)

	RAE on germination rate				RAE on root growth			
	*df*	% SS	*F*‐value	*p*‐value	*df*	% SS	*F*‐value	*p*‐value
*Lactuca sativa*								
VOC source (V)	3	36.7	16.8	***	3	2.9	10.9	***
Dose (D)	1	10.5	14.4	***	1	12.7	143.4	***
Substrate (S)	1	1.7	2.3		1	0.6	6.2	*
V × D	3	0.6	0.3		3	1.6	6.0	***
V × S	3	11.6	5.3	**	3	11.2	42.0	***
D × S	1	2.6	3.6		1	0.8	9.5	**
V × D × S	3	1.3	0.6		3	1.5	5.6	***
Residuals	48	35.0	0.6		1,282	68.7	5.6	***
*Linum strictum*								
VOC source (V)	3	42.3	26.1	***	3	31.7	75.4	***
Dose (D)	1	7.1	13.2	***	1	7.7	53.0	***
Substrate (S)	1	19.7	36.4	***	1	3.6	24.5	***
V × D	3	0.8	0.5		3	2.7	6.2	***
V × S	3	3.1	1.9		3	5.2	11.8	***
D × S	1	0.4	0.8		1	0.0	0.0	
V × D × S	3	0.6	0.4		3	0.6	1.3	
Residuals	48	25.9			332	48.5		

Note

*F*‐values and associated *p*‐values (* for *p* < 0.05, ** for *p* < 0.01 and *** for *p* < 0.001) are indicated.

Abbreviations: *df*, degrees of freedom; % SS, percentage of sums of squares.

#### 3.2.1 Seed germination

**Figure 3:**
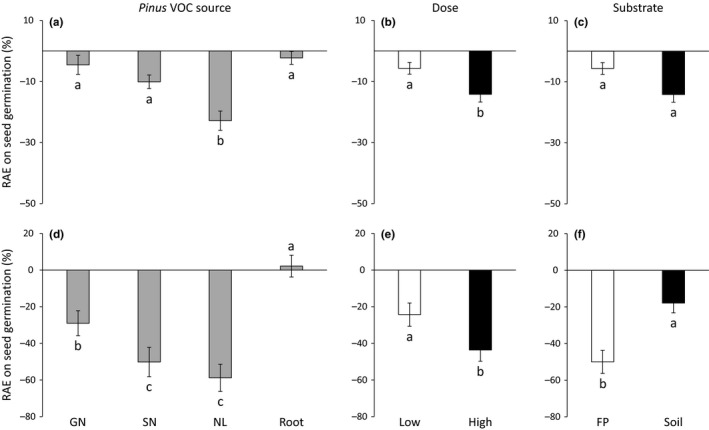
Relative allelopathic effect (RAE) on seed germination of *Lactuca sativa* (a, b, and c) and *Linum strictum* (d, e, and f) according to *Pinus halepensis* VOC source (a, d), dose (b, e) and substrate type (c, f). Values are mean ± *SE*. Different letters denote significant differences between treatments with a > b > c. Negative values of RAE indicate an inhibitory effect, whereas positive values indicate a stimulatory effect. GN, green needle; FP, filter paper; NL, needle litter; SN, senescent needle

**Figure 4:**
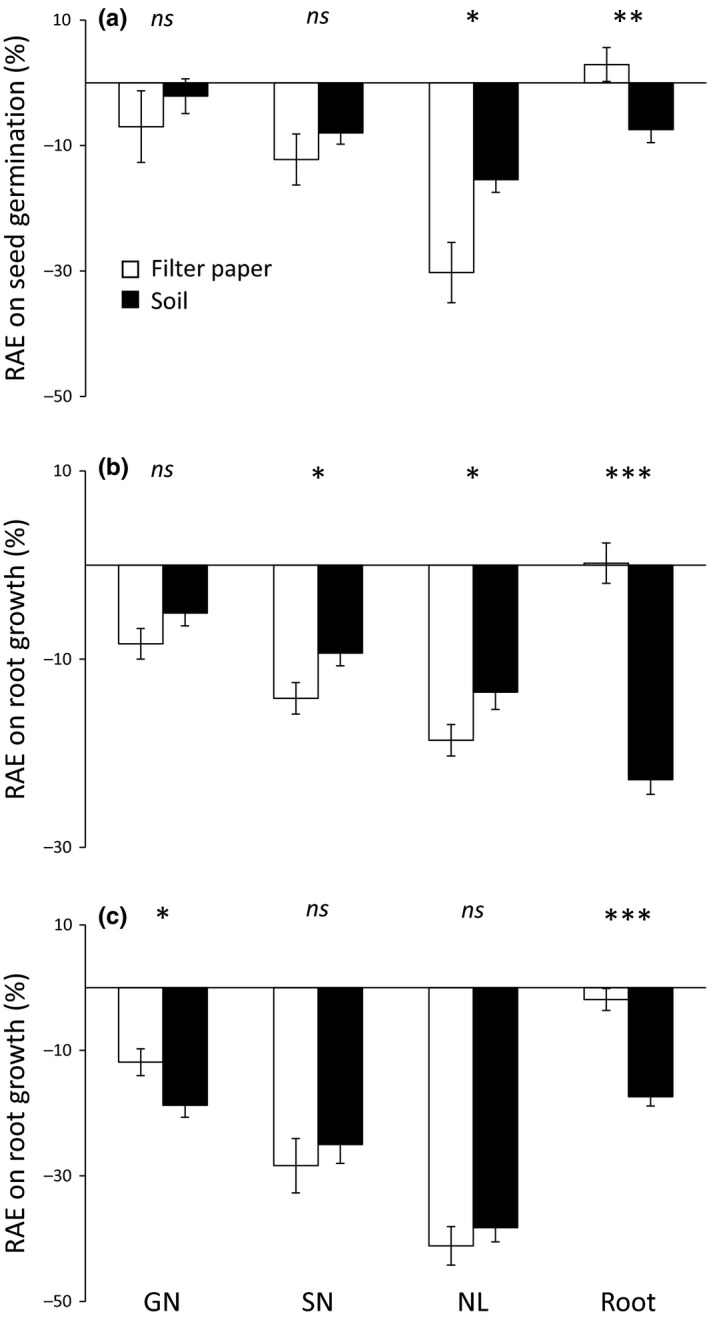
Relative allelopathic effect (RAE) on *Lactuca sativa* seed germination (a), *L. sativa* root growth (b), and *Linum strictum* root growth (c) according to the *Pinus halepensis* VOC source × substrate interaction (Table 2). Values are mean ± *SE*. Significant differences of RAE values between filter paper and natural soil are indicated with the respective symbols * for *p* < 0.05, ** for *p* < 0.01, *** for *p* < 0.001 and *ns* for *p* > 0.05. GN, green needle; NL, needle litter; SN, senescent needle

*Pinus* VOC source and dose had significant effects on seed germination (Table 2). Needle litter exhibited higher negative effects on seed germination than the three other VOC sources for both herbaceous target species (Figure 3a,d). Increasing dose reduced threefold and twofold seed germination of *L. sativa* and *L. strictum*, respectively (Figure 3b,e). The allelopathic effects on *L. strictum* seed germination were strongly reduced in natural soil compared to filter paper (Figure 3f). *Pinus* VOC source and substrate type interacted in their effects on *L. sativa* seed germination (Table 2): the negative effects of volatiles released from *Pinus* needle litter on *L. sativa* seed germination were reduced on natural soil compared to filter paper; by contrast, the effects of volatiles released from *Pinus* roots turned from positive into negative (Figure 4a).

#### 3.2.2 Root growth

**Figure 5:**
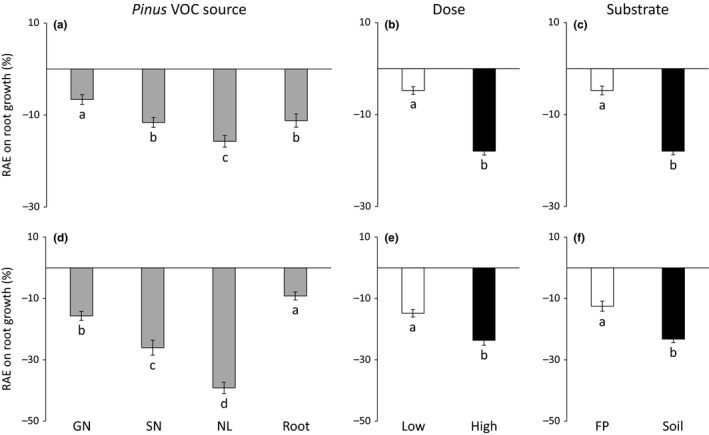
Relative allelopathic effect (RAE) on root growth of *Lactuca sativa* (a, b, and c) and *Linum strictum* (d, e, and f) according to *Pinus halepensis* VOC source (a, d), dose (b, e) and substrate type (c, f). Values are mean ± *SE*. Different letters denote significant differences between treatments with a > b > c. GN, green needle; FP, filter paper; NL, needle litter; SN, senescent needle

Allelopathic effects on root growth varied across needle physiological stages, with a clear trend to increasing negative effects according to the following order: green needle < senescent needle < needle litter (Figure 5a,d). In addition, root growth of both target species decreased with increasing dose (Figures 5b,e) and these negative effects were higher with natural soil compared to filter paper (Figure 5c,f). However, significant interactions between *Pinus* VOC source, dose, and substrate type were observed (Table 2).

As reported for *L. sativa* seed germination, *Pinus* VOC source and substrate type interacted in their effects on root growth for both target species (Table 2; Figure 4). The inhibitory effects of *Pinus* roots and green needles (only for *L. strictum*) were enhanced with natural soil compared to filter paper (Figure 4b,c). By contrast, the inhibitory effects of both senescent needles and needle litter on *L. sativa* root growth were reduced with natural soil compared to filter paper (Figure 4b).

**Figure 6:**
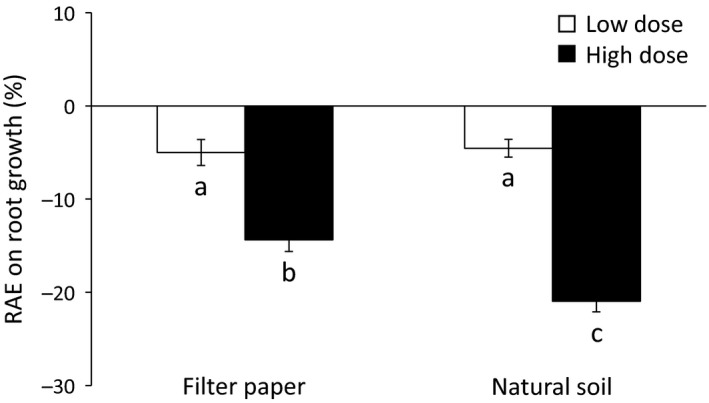
Relative allelopathic effect (RAE) on *Lactuca sativa* root growth according to the dose × substrate interaction (Table 2). Values are mean ± *SE*. Different letters denote significant differences between treatments with a > b > c

Regarding the significant dose × substrate type interaction on *L. sativa* root growth (Table 2), a similar inhibitory effect was observed at low dose on both filter paper and natural soil, while the inhibitory effect at higher dose was remarkably higher on natural soil compared to filter paper (Figure 6).

**Figure 7:**
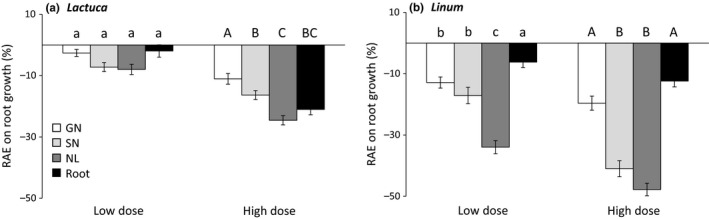
Relative allelopathic effect (RAE) on root growth of *Lactuca sativa* (a) and *Linum strictum* (b) according to the *Pinus halepensis* VOC source × dose interaction (Table 2). Values are mean ± *SE*. Different letters denote significant differences between VOC sources with a > b > c (lower case = low dose; upper case = high dose). GN, green needle; NL, needle litter; SN, senescent needle

Finally, the significant *Pinus* VOC source × dose interaction (Table 2) suggested that the range of allelopathic effects across the four *Pinus* VOC sources differed between low and high doses (Figure 7). A similar inhibitory effect between the four *Pinus* VOC sources on *L. sativa* root occurred at low dose, while higher inhibitory effects and a clear trend to increasing allelopathic effects with increasing needle physiological stage was observed with the higher dose (Figure 7a). Regarding *L. strictum*, senescent needles exhibited a similar inhibitory effect than green needles at low dose, while senescent needles exhibited a similar inhibitory effect than needle litter at high dose (Figure 7b).

### 3.3 Allelopathic bioassay with β‐caryophyllene

**Figure 8:**
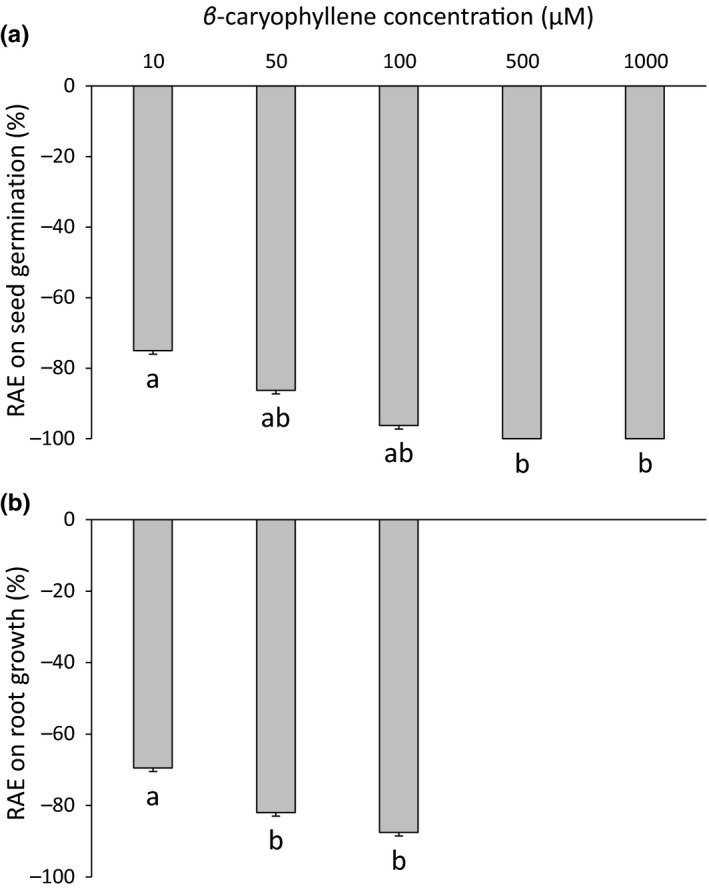
Relative allelopathic effects (RAE) of different β‐caryophyllene concentrations on seed germination (a) and root growth (b) of *Lactuca sativa*. Values are mean ± *SE*. Different letters denote significant differences between concentrations with a > b

β‐caryophyllene strongly inhibited seed germination and root growth of *L. sativa*. Seed germination was reduced by 75% at 10 µM and totally suppressed from 500 µM (*K *= 13.7, *p* < 0.01, Figure 8a). Root growth was reduced from 70% at 10 µM to 88% at 100 µM (*K *= 10.1, *p *< 0.01, Figure 8b).

## 4 DISCUSSION

As inhibited seed germination rate and seedling root growth imply a decrease in recruitment and survival of individuals, our results evidenced a strong potential control of *P. halepensis* VOC on the dynamics of herbaceous species populations. Two previous studies already highlighted an allelopathic effect of green needle and root leachates on *L. strictum* seed germination and seedling growth during laboratory bioassays (Fernandez et al., 2006, 2013). Floristic inventories performed by Fernandez et al. (2013) highlighted an important decrease in *L. strictum* abundance in the field when pines were present in their neighborhood. Thus, in addition to the high sensitivity of *L. strictum* to *P. halepensis* nonvolatile allelochemicals, our study suggests that *P. halepensis* VOC may also control *L. strictum* demographic parameters in the field.

Needle physiological stage was a key factor of the observed allelopathic effects in the present study as they explained a large part of RAE variance on both seed germination and root growth of the two target species (percentages of sums of squares, Table 2). Interestingly, in contrast with the trend reported by Nektarios et al. (2005) with the use of *P. halepensis* needle leachates, we observed a clear increasing inhibitory effect related to VOC according to the evolution of needle physiological stage (green needle < senescent needle < needle litter). Since the allelochemicals released in leachates belong usually to phenolics (Fernandez et al., 2009; Santonja, Le Rouzic, et al., 2018), the water solubility, and rapid leaching of these compounds, could explain the decreasing allelopathic potentialities along needle physiological stages (Chomel et al., 2014; Hashoum et al., 2017). Santonja, Baldy, et al. (2015) and Chomel et al. (2014) reported that 40% and 80% of phenolics disappeared after 2 and 6 months of *P. halepensis* needle litter decomposition, respectively, supporting the findings of Nektarios et al. (2005).

β‐caryophyllene was the main volatile released from *P. halepensis* needles and roots (Figure 2). Several previous studies showed that β‐caryophyllene is constitutively present in *P. halepensis* branches and litter and is naturally released in a large variety of growing conditions (Ormeño et al., 2009; Ormeño, Fernandez, Bousquet‐Mélou, et al., 2007; Ormeño, Fernandez, & Mévy, 2007). Ormeño et al. (2009) showed that β‐caryophyllene occurs within needle litter of *P. halepensis* in the field. Likewise, Ormeño, Fernandez, Bousquet‐Mélou, et al. (2007) reported β‐caryophyllene emissions from *P. halepensis* branches growing in six Mediterranean natural forest sites. Likewise, Ormeño, Fernandez, and Mévy (2007) reported that *P. halepensis* seedlings also synthesize and emit β‐caryophyllene. In the present study, this sesquiterpene exhibited strong inhibitory effects on both, seed germination and root growth of *L. sativa*, a finding in line with previous studies that suggested that this compound may act as allelochemical to influence neighboring plant growth (Kong, Hu, & Xu, 1999; Sanchez‐Muñoz, Aguilar, King‐Díaz, Rivero, & Lotina‐Hennsen, 2012; Wang, Pen, Zeng, Ding, & Xu, 2009). Wang et al. (2009) reported that β‐caryophyllene inhibited both, seed germination and seedling growth of *Brassica campestris* L. and *Raphanus sativus* L., given thus potential support for the successful invasion of *Mikania micrantha* Kunth in China. In addition, Kong et al. (1999) reported that β‐caryophyllene, by inhibiting seedling growth of several crop species (*Solanum Lycopersicon* L., *Raphanus sativus* L., and *Vigna radiate* (L.) R. Wilczek), could partly explain the strong allelopathic potentialities of the widespread weed *Ageratum conyzoides* L. in south China and Southeast Asia. Despite it is well known that allelopathic interactions are not due to a single compound but rather to a pool of several allelochemicals acting synergistically to inhibit or stimulate growth (Reigosa et al., 1999), the increasing release of β‐caryophyllene along needle physiological stages (Figure 2) could partly explain the increasing allelopathic effects along needle physiological stages in the present study. Other terpenes known as allelochemicals such as α‐pinene, 3‐carene, or limonene (Abrahim, Braguini, Kelmer‐Bracht, & Ishii‐Iwamoto, 2000; De Martino et al., 2010; Singh, Batish, Kaur, Arora, & Kohli, 2006) showed the same trend of increasing release as β‐caryophyllene (Appendix 1), giving additional support to increasing allelopathic effects according to needle physiological stages. However, we acknowledge that the volatile emission was measured only 1 hr after the pine material was put in the microcosm while this pine material remained in the microcosm for 2 days, suggesting that potentially other volatiles have been released by *P. halepensis* needles and roots during the experiment but not detected by the SPME fibers.

Laboratory bioassays using filter paper are frequently reported to overestimate the ability of allelochemicals to influence the germination and growth parameters of neighboring target plants (Fernandez et al., 2013). Indeed, the effects of allelochemicals are less inhibitory, disappear, or even become positive under natural soil (e.g., Fernandez et al., 2013; Hashoum et al., 2017) as microbial communities strongly influence the persistence, availability and biological activity of allelochemicals through volatile assimilation, degradation, and transformation (Blum & Shafer, 1988; Inderjit, 2005; Kaur et al., 2009). However, in the present study, numerous inhibitory effects observed with filter paper as a substrate were similar or amplified when using natural soil, highlighting that soil microorganisms are not necessarily able to limit the negative effect of VOC released from *P. halepensis* on herbaceous target species. This was particularly the case for the impact of VOC released from *P. halepensis* roots on seedling growth, suggesting that microbial degradation/transformation of these VOC could lead to degraded products with increased negative allelopathic effects. In addition, the impact of VOC released from green needles was enhanced, while those from senescent needles or needle litter were reduced with natural soil as substrate. We can speculate that a better ability of soil microorganisms to degrade the VOC released by senescent needles or needle litter leads to a reduction in their allelopathic effects as compared to those released by green needles. However, we acknowledge that we only used soil free from *P. halepensis* influence in the present study, that is, soil whose microbial community was not frequently in contact with the allelochemicals (phenolics and terpenes) released by *P. halepensis*. The soil microbial community under the influence of *P. halepensis* could be completely different and, as a result, could have an altered effect on the outcome of plant–plant chemical interaction mediated by *P. halepensis* VOC. These hypotheses would need new laboratory experiments specifically designed to study such microbial‐driven chemical transformations.

## 5 CONCLUSION

The present study confirms the strong allelopathic potentialities of *P. halepensis* as seed germination and seedling growth of the two target herbaceous species were mainly inhibited by VOC released from pine needles and roots. In addition, we demonstrated for the first time a clear increasing inhibitory effect of VOC according to the evolution of needle physiological stage (green needle < senescent needle < needle litter). Finally, our results pointed out that soil microorganisms are not necessarily able to limit the negative effect of VOC on herbaceous target species.

## CONFLICT OF INTEREST

None declared.

## AUTHOR CONTRIBUTIONS

MS, ABM, and CF designed the experiment. MS and SG performed the experiment. MS analyzed the data and led the writing of the manuscript. All authors contributed critically to the drafts and gave final approval for publication.

## Data Availability

Dryad https://doi.org/10.5061/dryad.s0b179p.

## APPENDIX 1

Composition of volatile organic compounds released from *Pinus halepensis* needles and roots.

**Table:**

Compounds	Exp RI	Lit RI	CAS number	Green needles		Senescent needles		Needle litter		Roots	
				Peak area	Percentage	Peak area	Percentage	Peak area	Percentage	Peak area	Percentage
Monoterpenes											
α‐Thujene	925	924	2867‐05‐2		Tr.	358,134	0.1	410,338	0.1		
α‐Pinene*	930	932	80‐56‐8	10,346,907	4.6	17,524,595	5.8	19,951,489	3.4	10,054,231	18.0
Sabinene	971	969	3387‐41‐5	1,572,906	0.7	6,618,945	1.8	10,598,025	1.8		Tr.
Myrcene*	990	988	123‐35‐3	36,174,796	16.0	28,934,513	7.0	40,881,515	7.0	465,899	0.8
3‐Carene*	1,006	1,008	13466‐78‐9	9,039,854	4.0	9,172,857	3.0	13,243,330	2.3	305,225	0.5
α‐Terpinene*	1,014	1,014	99‐86‐5	378,716	0.2	881,019	0.3	2,353,116	0.4		
p‐Cymene*	1,023	1,020	99‐87‐6	2,090,340	0.9	4,847,511	1.6	11,897,482	2.0	212,077	0.4
Limonene*	1,027	1,024	138‐86‐3	2,384,835	1.1	5,735,130	1.9	12,187,549	2.1	643,660	1.1
β‐Ocimene, cis‐	1,039	1,032	3338‐55‐4		Tr.		Tr.	501,366	0.1		
β‐Ocimene, trans‐	1,049	1,044	3779‐61‐1	1,134,531	0.5	2,726,094	0.9	4,706,409	0.8		
γ‐Terpinene*	1,058	1,054	99‐85‐4	1,433,499	0.6	2,779,971	0.9	6,473,793	1.1		
δ‐Terpinene	1,086	1,086	586‐62‐9	7,751,217	3.4	17,922,640	6.0	39,209,696	6.7	270,637	0.4
p‐Mentha‐1,3,8‐triene	1,106	1,108	18368‐95‐1			884,388	0.3	1,006,077	0.2		
p‐Mentha‐1,5,8‐triene	1,110	1,112	21195‐59‐5		Tr.	327,055	0.1	741,572	0.1		
Oxygenated monoterpenes											
Mentha‐2,8‐dien‐1‐ol	1,132	1,133	3886‐78‐0	152,253	0.1	491,005	0.2				
Camphor*	1,140	1,141	76‐22‐2	200,666	0.1	1,385,989	0.5	954,450	0.2		Tr.
3‐Pinanone, trans‐	1,156	1,158	547‐60‐4	461,983	0.2	523,886	0.2	1,386,736	0.2		
Pinocarvone	1,158	1,160	30460‐92‐5			455,894	0.2		Tr.		
3‐Pinanone, cis‐	1,168	1,172	15358‐88‐0	625,857	0.3	1,928,458	0.6	2,276,193	0.4		
4‐Terpineol*	1,174	1,174	562‐74‐3	812,368	0.4	3,588,528	1.2	2,901,480	0.5		
p‐Cymen‐8‐ol	1,181	1,179	1197‐01‐9		Tr.	596,384	0.2	422,419	0.1		
α‐Terpineol*	1,190	1,186	98‐55‐5	155,272	0.1	569,885	0.2	594,605	0.1		
Verbenone*	1,203	1,204	80‐57‐9	196,893	0.1	317,261	0.1	320,962	0.1	186,682	0.3
Monoterpene derivates											
Carvacrol, methyl ether	1,242	1,241	6379‐73‐3		Tr.	306,988	0.1	360,329	0.1		
Isoborneol, acetate	1,283	1,283	125‐12‐2			500,659	0.2	548,059	0.1		Tr.
Sesquiterpenes											
α‐Cubebene	1,348	1,345	31141‐66‐9	308,896	0.1	478,395	0.2	1,622,287	0.3		Tr.
Cyclosativene	1,364	1,369	30541‐92‐5	912,179	0.4	412,837	0.1	1,267,626	0.2	608,193	1.1
Copaene	1,374	1,374	13567‐62‐9	3,866,030	1.7	4,645,349	1.5	10,363,372	1.8	2,976,659	5.3
β‐Bourbonene	1,383	1,387	13828‐08‐5	198,267	0.1	256,843	0.1	987,030	0.2		Tr.
β‐Cubebene	1,388	1,387	23526‐21‐8	237,280	0.1	286,492	0.1	923,549	0.2	107,540	0.2
β‐Elemene	1,392	1,389	33880‐83‐0	223,101	0.1	550,915	0.2	1,287,407	0.2		Tr.
Longifolene*	1,402	1,407	475‐20‐7					426,933	0.1	1,485,812	2.6
Isocaryophyllene	1,408	1,408	61217‐74‐1	1,004,437	0.4	849,294	0.3	2,518,016	0.4		Tr.
β‐Caryophyllene*	1,417	1,417	17627‐40‐6	78,630,885	34.8	108,704,883	36.2	310,965,356	53.3	14,269,696	25.6
β‐Copaene	1,429	1,430	18252‐44‐3	1,062,919	0.5	2,303,947	0.8	5,304,121	0.9	1,505,045	2.7
α‐Guaiene	1,438	1,437	3691‐12‐1		Tr.	423,833	0.1	961,784	0.2		
Guaia‐6,9‐diene	1,443	1,442	37839‐64‐8	333,554	0.1	598,957	0.2	1,878,411	0.3		
α‐Himachalene	1,449	1,449	41702‐31‐2		Tr.	739,854	0.2			154,872	0.3
α‐Caryophyllene*	1,453	1,452	6753‐98‐6	15,738,469	7.0	20,909,453	7.0	663,457	0.1	2,456,448	4.4
Cadina‐1(6), 4‐diene, trans‐	1,474	1,475	931410‐54‐7		Tr.	484,498	0.2	589,353	0.1		
γ‐Muurolene	1,478	1,478	30021‐74‐0	958,492	0.4	2,379,018	0.8	2,421,011	0.4	876,675	1.6
Germacrene D	1,482	1,484	105453‐16‐5	199,903	0.1	274,159	0.1	888,134	0.2	473,752	0.8
β‐Selinene	1,492	1,489	21488‐94‐8	369,225	0.2	516,694	0.2	2,314,984	0.4		
α‐Muurolene	1,502	1,500	31983‐22‐9	5,890,566	2.6	5,269,095	1.8	5,470,007	0.9	12,505,286	22.3
α‐Farnesene, trans, trans‐	1,510	1,505	502‐61‐4	245,532	0.1	504,495	0.2	549,898	0.1	168,261	0.2
γ‐Cadinene	1,516	1,513	39029‐41‐9	295,765	0.1	745,112	0.2	484,686	0.1		Tr.
δ‐Cadinene	1,524	1,522	60305‐17‐1	2,273,959	1.0	1,615,905	0.6	3,872,377	0.7	812,058	1.4
Cadina‐1,4‐diene	1,535	1,533	16728‐99‐7	183,093	0.1	242,526	0.1	449,617	0.1		
Oxygenated sesquiterpenes											
Caryophyllene oxide*	1,585	1,582	1139‐30‐6	10,874,006	4.8	8,156,453	2.7	10,138,100	1.7	1,643,386	2.9
Humulene oxide II	1,613	1,608	67737‐67‐1	1,610,896	0.7	1,375,880	0.5	1,311,156	0.2	249,478	0.4

Abbreviations: CAS Number, Chemical Abstracts Service Number; Exp RI, Experimental Retention Index calculated with the formula of van Den Dool and Kratz (1963); Lit RI, Literal Retention Index based on Adams (2007); Peak area, peak area of the compound in the chromatogram; Percentage, peak surface percentage in the chromatogram; Tr., Traces (peak surface percentage < 0.1%).

* Identification confirmed by commercial standard coinjection.
